# Editorial: Molecular and cytogenetic research advances in human reproduction - volume II

**DOI:** 10.3389/fendo.2023.1232953

**Published:** 2023-07-17

**Authors:** Xiufeng Chu, Ihtisham Bukhari, Rick Francis Thorne, Qinghua Shi

**Affiliations:** ^1^ Henan Key Laboratory of Helicobacter pylori, Microbiota and Gastrointestinal Cancers, Marshall Medical Research Center, Fifth Affiliated Hospital of Zhengzhou University, Zhengzhou, China; ^2^ Translational Research Institute, Henan Provincial and Zhengzhou City Key Laboratory of Non-coding RNA and Cancer Metabolism, Henan International Join Laboratory of Non-coding RNA and Metabolism in Cancer, Henan Provincial People’s Hospital, Academy of Medical Sciences, Zhengzhou University, Zhengzhou, Henan, China; ^3^ School of Basic Medical Sciences, Division of Life Sciences and Medicine, Biomedical Sciences and Health Laboratory of Anhui Province, Institute of Health and Medicine, Hefei Comprehensive National Science Center, University of Science and Technology of China, Hefei, China

**Keywords:** male infertility, reproduction disorders, female infertility, infertility treatment and complications, genetic infertility

Infertility is a prevalent disorder that affects over 15% of couples worldwide, with various causes such as meiotic arrest due to cytogenetic shuffling, toxins, and other physical factors (1–4). Among the genetic causes of male infertility, chromosomal abnormalities have been reported in approximately 6% of cases, and microdeletions in the Y chromosome account for spermatogenic failure in 5-20% of men with azoospermia (3, 5–8). The most common structural Y chromosome aberrations in infertile men with azoospermia have been reported to be an isodicentric or isochromosome Y, with duplications in the Yp region and deletions in the Yq region, or vice versa (9–12). The human X and Y chromosomes pair at their distal short arms during male meiosis, with the telomeres first approaching each other during the zygotene stage of meiotic prophase (13, 14). As pachytene progresses, a paired segment is formed, and a synaptonemal complex (SC) which, on average, extends to about one-third of the total length of the Y chromosome (15–17).

Recently, pathogenic genetic mutations have been identified in individuals suffering from reproductive disorders; these mutations are generally detected in the genes mainly involved in germ cell development and other reproductive processes (18–21). Nevertheless, thousands of functionally relevant genes are expressed in human testes and ovaries, and malfunctioning of even a single gene can potentially cause infertility (22–24). Although the overall mechanistic contribution of specific genes has been studied in animal models, more attention is now being paid to clinically detected alterations in DNA, which include mutations potentially affecting gene regulatory sequences such as the 3’UTR (25, 26). However, the impact of mutations in gene 3’UTR sequences may not be adequately recognized in current sequencing analyses. This provides a potentially massive gap between fertility genetics and clinical applications, a problem which needs to be addressed to ensure proper diagnosis and treatment of reproductive disorders.

The objective of this Research Topic was to gather clinical and basic research studies that report on cytogenetic, molecular, and clinical aspects of human reproduction. After rigorously reviewing submitted articles, the current volume presents an authoritative collection of eleven articles exploring new dimensions of human reproduction, providing valuable insights and advancing our understanding in this field.

Bourdon et al. conducted a study to evaluate the putative role of FGF21 on spermatozoan function. The results showed that *in vitro* treatment by FGF21 significantly increased sperm motility and ATP levels, indicating that the metabolic factor FGF21 positively modifies the activity and quality of human spermatozoa parameters. This finding has implications for developing therapeutic strategies for male infertility.

Li et al. conducted a comprehensive investigation into the association between blastocyst morphology, developmental rate, euploidy and live birth rates (LBRs) in single euploid frozen-thawed embryo transfer (FET) cycles. Their results demonstrated a positive association between the quality of embryos, blastocyst developmental rate, and euploidy rate in middle-aged women, which was positively associated with LBRs. These findings provide insights into the understanding of embryonic development and potentially have significant implications for improving assisted reproductive technology (ART) outcomes.

Liu et al. determined the association between antinuclear antibodies (ANAs) and recurrent pregnancy loss (RPL) and the effects of immunotherapy on pregnancy outcomes in women with positive ANAs and history of RPL. Their findings suggest that the presence of ANAs is strongly correlated with RPL and has prognostic value for subsequent pregnancy outcomes in women with a history of recurrent pregnancy loss. This study suggests ANAs levels could be considered in the diagnostic workup of RPL and the potential benefit of immunotherapy in improving pregnancy outcomes for these patients.

Ma et al. conducted a comprehensive review of the factors associated with recurrent implantation failure (RIF), which can be caused by a range of factors such as immunology, thrombophilias, endometrial receptivity, microbiome, anatomical abnormalities, male characteristics, and embryo aneuploidy. The authors suggest that targeted and precision therapy can improve the chances of successful implantation in RIF patients, indicating the need for personalized treatment strategies for this patient population. This study sheds light on the complex nature of RIF and highlights the importance of understanding its underlying mechanisms for developing practical treatment approaches.

Chuang et al. conducted a study that analyzed the correlation of mitochondrial DNA (mtDNA) content of a single biopsy at trophoblast with the developmental potential and reproductive outcomes of the blastocyst. The authors concluded that the mtDNA ratio depends on the period after blastocyst formation. Lower mtDNA ratios were observed at day 6 of euploid single embryo transfers, suggesting that the timeline of the embryos is an important covariate of mtDNA content. These findings have implications for optimizing the selection of embryos for transfer and improving outcomes in assisted reproductive technology (ART).

Wu et al. conducted a retrospective study investigating the correlation between transferred embryos and multiple pregnancy/live birth rates in frozen embryo transfer cycles. Their findings suggest that single-good-quality blastocyst transfer is an appropriate strategy for women under 40 years old. In contrast, double high-quality embryo transfer may be more suitable for women over 40. This study provides valuable information for clinicians in selecting embryos for transfer and improving the chances of successful pregnancy outcomes in women undergoing frozen embryo transfer.

Yang et al. conducted a study that found that the Sperm DNA Fragmentation Index was positively correlated with blastocyst aneuploidy rate, indicating that sperm DNA damage may contribute to chromosomal abnormalities in embryos. On the other hand, sperm motility and morphology rate were negatively correlated with blastocyst aneuploidy rate, suggesting that better sperm quality is associated with improved embryonic chromosomal integrity. These findings highlight the importance of assessing sperm quality when selecting embryo transfer in ART procedures.

Liu et al. found that the rate of *de novo* chromosomal abnormalities increased with maternal or paternal age. However, controlled ovarian hyperstimulation parameters did not influence the incidence of *de novo* chromosomal abnormalities or clinical pregnancy outcomes. These findings suggest that while advanced maternal or paternal age poses a risk for chromosomal abnormalities, factors related to ovarian stimulation protocols may not significantly impact the incidence of chromosomal abnormalities or pregnancy outcomes. These findings have implications for patient counseling and treatment planning in ART procedures.

Yuan et al. demonstrated that sperm telomere length has diagnostic and predictive value for male fertility and clinical pregnancy and may be used as a biomarker for the diagnosis of male infertility and predicting embryonic development. This finding has implications for improving the accuracy of male infertility diagnosis and developing personalized treatment plans for couples undergoing assisted reproduction.

Racca et al. aimed to determine the ideal progesterone (P4) levels for the day of embryo transfer and whether supplementing progesterone (P4) on the day of human chorionic gonadotropin (hCG) release can improve the success rate of frozen embryo transfer (FET) cycles. They retrospectively analyzed 664 females who had vaginal 600 mg/day of P4 as hormone replacement therapy (HRT)-FET cycles starting 6 days before the FET. The study found that the likelihood of detecting P4-hCG < 10.6 ng/ml decreased as the level of serum P4 the day before ET increased, indicating the importance of adequate progesterone supplementation in ART cycles. Additionally, the study showed that low P4 levels if not supported by supplementation were associated with significantly lower live birth rates (LBR), suggesting the need for careful monitoring of P4 levels and timely intervention in ART cycles.

Ruan et al. identified biallelic mutations of CEP70 in two unrelated infertile male individuals with oligoasthenoteratozoospermia that followed a recessive inheritance pattern. Furthermore, the study found morphological and ultrastructural defects in the acrosome and flagellum of sperm from the patient, which were similar to those seen in Cep70-/- male mice. These findings provide insights into the genetic basis of male infertility and highlight the importance of investigating rare genetic variants in diagnosing and managing infertility.

## Conclusion

Recent advances in next-generation sequencing (NGS) technologies have transformed the landscape of investigating rare and common human disorders. The ability to generate genome-wide sequencing data with in-depth coverage in a short time frame represents a cost-effective replacement for conventional approaches that primarily focus on specific regions for gene discovery and clinical testing. The articles included in the current Research Topic provide valuable insights into various aspects of human reproduction, including the roles of different genes, hormones, and technologies. These findings have significant implications for improving our understanding of reproductive biology and developing more effective diagnostic and therapeutic strategies for individuals affected by infertility.

## Author contributions

All authors listed have made a substantial, direct, and intellectual contribution to the work and approved it for publication.
